# Case report and literature review: Golidocitinib as a potential treatment for monomorphic epitheliotropic intestinal T-cell lymphoma

**DOI:** 10.3389/fonc.2026.1729386

**Published:** 2026-02-18

**Authors:** Xinlong Xu, Chang Dong, Jiashuo Guo, Xiaolin Chang, Ruirui Yuan, Yu Zhang, Shuting Gou, Liying Xue, Jie Li

**Affiliations:** 1 Graduate School, Hebei North University, Zhangjiakou, China; 2 Department of Hematology, Hebei General Hospital, Shijiazhuang, China; 3 Department of Pathology, Hebei Medical University, Shijiazhuang, China

**Keywords:** case report, diagnosis, Golidocitinib, JAK-1 inhibitor, monomorphic epitheliotropic intestinal T-cell lymphoma

## Abstract

Monomorphic epitheliotropic intestinal T-cell lymphoma (MEITL) is a rare type of non-Hodgkin lymphoma and a highly aggressive malignancy with a median overall survival of 7 months. Meanwhile, owing to the complex and confusing symptoms including nonspecific gastrointestinal symptoms, systemic manifestations and additional clinical manifestations associated with the extraintestinal involvement organs, MEITL is often diagnosed at an advanced stage. We report three cases of MEITL, and searched PubMed indexed articles between 2016 and 2024 for these terms: “MEITL” and “case report”. We found that younger patients, those with intestinal perforation, and those who did not receive chemotherapy had worse prognoses. Importantly, we found that treatment with the JAK-1 inhibitor Golidocitinib in combination with the GDP regimen (Gemcitabine, Dexamethasone and Cisplatin) can improve patient prognosis. The study suggests that Golidocitinib could potentially improve the prognosis of MEITL patients by targeting the JAK-STAT pathway, although further validation of its long-term effectiveness is needed.

## 1 Introduction

Monomorphic epitheliotropic intestinal T-cell lymphoma (MEITL), originating from intraepithelial T-cells in the gastrointestinal tract, is an extremely rare type of non-Hodgkin lymphoma (1). In another recent international study, enteropathy-associated T-cell lymphoma (EATL) accounts for approximately 5.4% of all peripheral T-cell lymphomas (PTCL), with a higher incidence in Europe and North America (2). Statistically, the overall incidence of EATL is 0.5–1 per million (3). MEITL, named the EATL type II before 2016, accounts for 10–20 percent of EATL (4). In 2016, the World Health Organization (WHO) redefined EATL Type II without celiac disease as MEITL, which affects Hispanics, Asians, and middle-aged and older men (1).

Patients with MEITL generally present with nonspecific gastrointestinal symptoms, including abdominal pain, nausea, vomiting, bloating and diarrhea (2). Systemic manifestations such as weight loss and fatigue are also common. When extraintestinal involvement occurs, affecting organs such as the brain and lungs, patients exhibit additional clinical manifestations associated with the affected systems (5). The nonspecific nature of these symptoms often leads to initial misdiagnosis as common intestinal disorders, such as enteritis, resulting in delayed diagnosis and treatment. Importantly, MEITL is a highly aggressive malignancy, with the median overall survival of only 7 months (4). Therefore, timely diagnosis and intervention are crucial for patient management. Currently, the diagnosis for MEITL is made mainly according to cellular morphology of tumor tissue and its immunophenotype, being CD3+, CD4-, CD8+, CD56+ (although rare cases with negative CD8 or CD56 have been reported) (4). The most common site of MEITL is the small intestine, followed by the rest of the digestive tract, such as the large intestine and stomach (2, 6, 7). Clinically, it is extremely difficult for most patients to get a definite diagnosis in a timely manner, due to difficulty obtaining biopsy samples through gastroscopy and colonoscopy.

Currently, there is no standardized treatment protocol for MEITL established by the WHO. At present, there are various treatment methods for MEITL, including chemotherapy, surgery, and hematopoietic stem cell transplantation (HSCT). However, the overall effectiveness of these treatment methods is suboptimal. And further research is needed on the selection of chemotherapy drugs and the timing of surgical resection.

## 2 Methods

Herein, we report the diagnostic process, the treatment approach, and follow-up results of three cases of MEITL. In Case 3, the patient was treated with Golidocitinib, marking its first reported use for this condition, and achieved a partial response (PR). Furthermore, we conducted a retrospective analysis of data from 62 reported MEITL patients between 2016 and 2024, focusing on initial symptoms, diagnosis process, and treatment modalities. This study aims to provide valuable insights to facilitate the early detection, diagnosis, and treatment of MEITL.

## 3 Cases descriptions

Three male patients were admitted to the Department of Hematology of Hebei General Hospital (Shijiazhuang) from 2020 to 2024 because of different symptoms. The characteristics of these cases were summarized in **Table 1**.

**Table 1:** The diagnosis and treatment of MEITL patients.

Patient	Gender, age(years)	Symptoms	Complication	Location	Methods	Immune phenotype	Treatment	Follow-up
Case1	Male, 78	Limb weakness and abdominal pain	Intestinal perforation	Small intestine	Surgery	CD3(+), CD8(+), CD56(+), CD7(+), TIA-1(+), GranzymeB(+), Ki-67 (Active zone 90%), CD4(-), CD5(-),CD20(-), CD21(-), EBER(-)	COP*1, Rituximab	3 months, death
Case2	Male,51	Abdominal pain and diarrhea	Intestinal obstruction	Small intestine	Small bowel endoscope	CD3(+), CD8(+), CD56(+), CD4(+), TIA-1(+), GranzymeB(+), Ki-67 (Active zone 90%), CD20(-), EBER(-)	CHOP*2	6 months, death
Case3	Male, 62	Diarrhea and hematochezia	Intestinal obstruction	Small intestine, colon	Surgery	CD3(+), CD8(+), CD56(+),TIA-1(+), GranzymeB(+), Ki-67 (Active zone 40%), CD4(-), CD20(-), CD21(-), EBER(-)	CHOP and Chidamide*3, Golidocitinib and GDP	Alive

### 3.1 Case 1

A 78-year-old male with a 14-month history of lymphoma presented to our hospital complaining of intermittent limb weakness and abdominal pain of over one month. Fourteen months prior, the patient had been diagnosed with lymphoma following findings of multiple enlarged lymph nodes in the small intestine by computed tomography (CT) and positron emission tomography-computed tomography (PET-CT) (**Supplementary Figure S1**). The patient was subsequently treated with one cycle of COP (Cyclophosphamide, Vincristine, and Prednisone) chemotherapy but refused further treatment due to a history of coronary heart disease and cerebral infarction. Due to progressively worsening abdominal pain, the patient required readmission for a comprehensive clinical assessment. Laboratory tests revealed mild anemia and elevated inflammatory markers (hemoglobin: 101 g/L; C-reactive protein: 63.81 mg/L) (**Supplementary Table S2**). CT imaging demonstrated significant thickening of the intestinal wall at the lesion site compared to the study from 14 months prior. Based on these findings, the patient was considered for progressive intestinal lymphoma and treated with a single dose of Rituximab (0.6 g). Ten days later, the patient developed intestinal perforation, indicated by CT scan, and subsequently underwent emergent surgery. Numerous tumor cells were identified within the lesional tissue as revealed by Hematoxylin-Eosin (HE) staining (**Supplementary Figure S1**). The immunophenotype of the lesion tissue showed CD3+, CD8+, CD56+, CD4-, CD5- (**Supplementary Figure S1**). Thus, the patient was clearly diagnosed with MEITL. However, due to disease progression and overall poor clinical condition, the patient was unable to tolerate systemic chemotherapy and instead received supportive care. One month later, the patient succumbed to multiple organ failure due to severe infection.

### 3.2 Case 2

A 51-year-old male was admitted to our hospital with a one-month history of abdominal pain and diarrhea. Fifteen days prior to admission, the patient was diagnosed as having multiple polyps of the colon using gastroscopy and colonoscopy at another hospital and was untreated. On admission, a comprehensive clinical assessment was conducted. Laboratory results indicated moderate anemia (**Supplementary Table S2**). Abdominal CT revealed incomplete intestinal obstruction and multiple enlarged lymph nodes (**Supplementary Figure S2**). Subsequent PET-CT imaging revealed multiple hypermetabolic foci within the small intestine and lymph nodes (**Supplementary Figure S2**). Histopathological analysis of biopsy samples obtained via small bowel endoscopy confirmed a T-cell lymphoma with an immunophenotype of CD3+, CD8+, CD56+, CD4-, CD5- (**Supplementary Figure S2**), suggesting a diagnosis of MEITL. Following diagnosis, the patient was initiated on the CHOP regimen (cyclophosphamide, doxorubicin, vincristine, and prednisone). Following two treatment cycles, abdominal CT imaging demonstrated stable tumor dimensions and a mild improvement in intestinal symptoms. However, the patient refused to continue chemotherapy due to poor general condition and economic factors, and unfortunately died 5 months later.

### 3.3 Case 3

**Figure 1:**
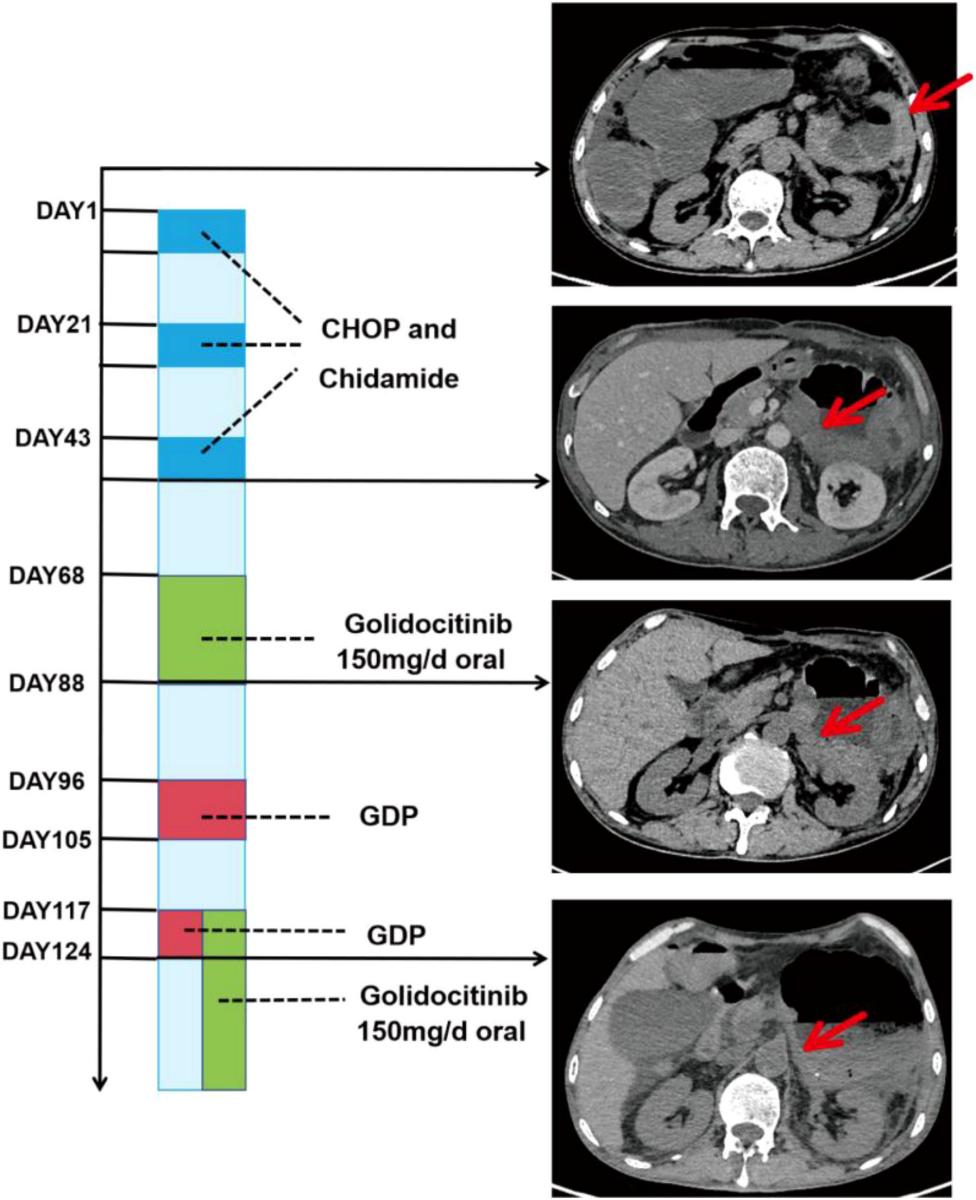
Treatment timeline and corresponding CT imaging changes for Case 3.

A 62-year-old male was admitted due to persistent diarrhea for more than 10 days and one day of hematochezia. Abdominal CT showed a malignant tumor in the small intestine and surgical resection was performed in the surgical department. Postoperative pathological examination of the resection specimen confirmed the diagnosis of MEITL, with an immunophenotype of CD3+, CD8+, CD56+, CD4- (**Supplementary Figure S3**). While recovering and awaiting initiation of systemic chemotherapy, the patient developed an internal abdominal hernia 22 days after the first surgery and underwent emergency surgery again. A post-operative PET-CT scan then revealed hypermetabolic lesions in the colon, indicative of residual or metastatic disease (**Supplementary Figure S3**). After sufficient recovery, the patient received three cycles of combined therapy with the CHOP regimen and Chidamide. Peripheral blood monitoring during this period showed no evidence of hematological involvement. However, a follow-up abdominal CT scan indicated disease progression. The treatment strategy was subsequently altered to include the JAK-1 inhibitor Golidocitinib and the GDP regimen (gemcitabine, dexamethasone, and cisplatin). A subsequent abdominal CT scan demonstrated a significant improvement in the patient’s condition compared to prior imaging. The timeline of therapeutic interventions and corresponding clinical responses is summarized in figures below (**Figure 1**). The patient remains under active treatment and monitoring in the hospital.

## 4 Discussion

MEITL, predominantly located in the gastrointestinal tract, is an extremely rare hematological malignancy, accounting for approximately 0.5% -1.6% of total non-Hodgkin lymphomas (2, 8, 9). Based on distinct immunophenotypic features relative to EATL, MEITL was classified as an independent T-cell lymphoma by the WHO in 2016 (1, 10). MEITL exhibits a poor prognosis, with the median overall survival of only 7 months (4). Early diagnosis and treatment may improve the survival of MEITL patients. However, it is extremely difficult to obtain a definitive diagnosis timely, due to the difficulty of obtaining adequate biopsy tissue. In this study, we report three cases of MEITL admitted to our hospital between 2020 and 2024.

The three patients presented with varying initial symptoms, including weakness, diarrhea, and hematochezia. Case 1 and Case 3 underwent emergency surgery for gastrointestinal perforation and malignant tumor, respectively, while Case 2 underwent biopsy via small bowel endoscopy.

All three cases were confirmed as MEITL based on immunohistochemical profiles (CD3+, CD8+, CD56+) in combination with clinical history. We also comprehensively reviewed the English literature about MEITL between 2016 and 2024, and summarized the clinical features of 62 patients including 3 cases in this case report. Among these cases, 87% of the patients had small bowel involvement, with 44 cases having involvement confined solely to the small bowel. Clinically, MEITL patients generally present with nonspecific gastrointestinal symptoms, including abdominal pain, weight loss, and diarrhea, and additional manifestations associated with the affected systems when extraintestinal involvement occurs (5). Due to these nonspecific symptoms, MEITL patients often overlook their condition in the early stages. Most patients seek medical attention only when complications such as intestinal perforation or obstruction arise.

In the diagnosis of MEITL, despite the introduction of PET-CT, definitive diagnosis still relies on immunohistochemical markers. For instance, Dazhi Kawamura et al. reported a case in which PET-CT revealed FDG uptake in the small bowel and surrounding lymph nodes, prompting surgical intervention for further evaluation and diagnosis (12). In a case reported by Sotaro Ozaka, tumor cells were detected only after multiple colonoscopies (13), while Marco et al. described a case in which the patient remained undiagnosed despite multiple gastric and colonoscopies, ultimately requiring small bowel resection for definitive diagnosis (14). Furthermore, a literature review highlighted the challenges of diagnosing intestinal non-Hodgkin lymphoma through endoscopy, including biopsy (15). However, obtaining samples via intestinal endoscopy poses significant challenges for MEITL patients due to technical limitations, limited patient tolerance, and economic constraints, making definitive diagnosis difficult. Based on previously reported data, we found that 81% of patients were diagnosed through surgery, while only 19% were diagnosed via endoscopy. Unfortunately, patients with intestinal perforation had a significantly lower survival rate beyond 12 months (27.3%) compared to those without this complication (68.2%). In particular, patients aged <55 had a significantly lower survival rate of only 20.0% beyond 12 months than those aged ≥55 years (**Table 2**). Accordingly, MEITL is a rare and aggressive lymphoma with a poor prognosis, often diagnosed at an advanced stage due to nonspecific symptoms and diagnostic challenges. Younger age (<55 years) and complications such as intestinal perforation were associated with worse outcomes.

**Table 2:** The relationships between clinical characteristics and the survival time in MEITL.

Characteristics	Total	Survival time		*P*
		≤12 months	>12 months	
Age(years)				
≥55	41	17 (41.5%)	24 (58.5%)	
<55	15	12 (80.0%)	3 (20.0%)	0.011
Sex				
Male	40	22 (55.0%)	18 (45.0%)	
Female	16	7 (43.8%)	9 (56.2%)	0.447
Location				
Small intestine	40	22 (55.0%)	18 (45.0%)	
The others	16	7 (43.8%)	9 (56.2%)	0.447
Diagnostic method				
Endoscope	11	4 (36.4%)	7 (63.6%)	
Surgical operation	45	25 (55.6%)	20 (44.4%)	0.253
Clinical manifestation				
Loss of weight	10	4 (40.0%)	6 (60.0%)	
The others	27	12 (44.4%)	15 (55.6%)	0.809
Complication				
Intestinal Perforation	22	16 (72.7%)	6 (27.3%)	
The others	22	7 (31.8%)	15 (68.2%)	0.007
Chemotherapy				
No	6	6 (100.0%)	0 (0%)	
Yes	42	19 (45.2%)	23 (54.8%)	0.012
Chemotherapy regimen				
Single regimen	29	13 (44.8%)	16 (55.2%)	
Polychemotherapy or Combination	13	6 (46.2%)	7 (53.8%)	0.936

incomplete records were omitted from the final analysis to ensure statistical validity.

In terms of treatment, the consensus for MEITL does not exist. Some studies have indicated that surgical treatment can extend survival, but recurrence often occurs postoperatively (7). Currently, the majority of patients receive chemotherapy regimens, such as CHOP (24%), CHOPE (cyclophosphamide, doxorubicin, vincristine, prednisone, and etoposide) (20%), and even a combination of several regimens (21%) (**Table 2**). A MEITL patient was reported to achieve complete remission (CR) after three cycles of CHOP and pralatrexate (16). Another report described a MEITL patient who survived for 6.5 years following treatment with a multi-modal regimen that included chemotherapy (CHOPE, GDP), autologous HSCT, small bowel resection, and the anti-CCR4 antibody mogamulizumab (17). Chen reported that a 54-year-old MEITL patient received three cycles of a pegylated liposomal doxorubicin (PLD)-containing CHOPE regimen and achieved CR, remaining disease-free during a 15-month follow-up (11).

Besides that, HSCT has been reported to be an alternative to intensive chemotherapy. Five MEITL patients who underwent HSCT after achieving complete or partial remission attained an overall survival of 26–67 months (4). Case reports have also documented long-term survival following HSCT. For instance, one Japanese patient remained disease-free for over 6 years (17), and a Chinese patient achieved 70 months of progression-free survival (7). Another patient also maintained a favorable outcome for 57 months, after treated with allogeneic HSCT (18). However, Min reported that among seven MEITL patients received upfront HSCT, four patients on autologous HSCT relapsed and died, whereas the other three patients receiving allogeneic HSCT maintained a CR by the final follow-up (19). In Chen’s retrospective cohort of 20 cases, 3 patients receiving autologous HSCT showed the survival of only 6–10 months, similar with patients without receiving autologous HSCT (20). Taken together, although the prognostic benefits of HSCT for patients remain unclear, it may be considered as an alternative to intensive chemotherapy with or without novel agents.

In this study, Case 1 only received supportive care owing to extremely poor physical condition and severe infection, whereas Case 2 received two cycles of CHOP, and then refused to continue chemotherapy due to economic burden. Regrettably, both MEITL patients died several months after discharge. In addition, we analyzed previously reported data and found that patients who received chemotherapy had a significantly higher survival rate (54.8%) than those who did not receive chemotherapy, who had a 0% survival rate beyond 12 months. Our results is consistent with the Tse cohort study reporting a 46% ORR (12/26) for chemotherapy, with rates of 42% for anthracycline-containing and 50% for non–anthracycline-containing regimens (4). Collectively, these findings suggest that active treatment confers a survival benefit, despite the lack of a standardized chemotherapy regimen.

In this study, it is noteworthy that after showing no response to three cycles of CHOP combined with Chidamide, Case 3 was switched to JAK-1 inhibitor Golidocitinib and GDP treatment. Golidocitinib, a highly selective JAK-1 inhibitor, demonstrates an ORR of approximately 40% in the treatment of relapsed or refractory PTCL (21, 22). The mutation of STAT5B, a downstream substrate of JAK-1, has been identified in 36.8% (7/19) of MEITL (EATL II) patients (23). In 9 Chinese MEITL cases, STAT5B (4/9), TP53 (4/9), and JAK-3 (3/9) were the most frequent mutations, implicating JAK-STAT pathway dysregulation as a key molecular feature of MEITL (20). Importantly, an *in vitro* study has demonstrated that the JAK inhibitor AZD1480 effectively inhibits STAT3/STAT5B mutant transduced cells (23). These findings suggest that targeting the JAK-STAT pathway may represent a promising therapeutic strategy for MEITL. Following Golidocitinib and GDP combination therapy, Case 3 demonstrated significant tumor regression with disease control achieved. Most recently, Golidocitinib was also treated combined with chemotherapy for PTCL patients, with an ORR of 80.0% and a CR rate of 73.3% (24). Furthermore, a multicenter phase 2 study of 7 MEITL patients showed an ORR of 85.7% and a CR rate of 71.4% with Golidocitinib and CHOP treatment (25).

This suggests the potential efficacy of Golidocitinib and GDP in MEITL treatment, although the long-term effects are unclear. However, the progression of Case 3 during follow-up underscores the challenges posed by comorbidities and the need for comprehensive management strategies in MEITL. To our knowledge, this represents the first reported use of Golidocitinib in the treatment of MEITL.

## 5 Conclusion

In summary, MEITL is often diagnosed at an advanced stage due to nonspecific symptoms and diagnostic challenges. Early diagnosis and active treatment, including chemotherapy and targeted therapies like Golidocitinib, may improve patient survival. However, the lack of a unified treatment regimen and the impact of complications such as intestinal perforation highlight the need for further research and comprehensive management strategies to enhance outcomes for MEITL patients. The first reported use of Golidocitinib in MEITL treatment shows promise but requires further investigation to determine its long-term efficacy.

## Data Availability

The original contributions presented in the study are included in the article/**Supplementary Material**. Further inquiries can be directed to the corresponding authors.
