# Supplementary material for: Case report and literature review: Golidocitinib as a potential treatment for monomorphic epitheliotropic intestinal T-cell lymphoma
Source: Front Oncol. 2026 Feb 18;16:1729386. doi: 10.3389/fonc.2026.1729386 (PMC12956697; doi:10.3389/fonc.2026.1729386)
Supplement: Supplementary file 1 [file DataSheet1.docx]

**Table S1 Clinical characteristics of MEITL patients**

| Characteristic | All patients (n=62) |
| --- | --- |
| Age(years) |  |
| ≥55 | 74%(46/62) |
| <55 | 26%(16/62) |
| Sex |  |
| Male | 71%(44/62) |
| Female | 29%(18/62) |
| Clinical manifestation |  |
| Abdominal pain | 74% (46/62) |
| Diarrhoea | 20%(12/62) |
| Loss of weight | 34%(21/62) |
| Diagnostic method |  |
| Endoscope | 19%(12/62) |
| Surgical operation | 81%(50/62) |
| Complication |  |
| Intestinal Perforation | 45%(28/62) |
| Intestinal obstruction | 13%(8/62) |
| Ulcer | 13%(8/62) |
| No | 29%(18/62) |
| Chemotherapy |  |
| CHOP | 24%(15/62) |
| CHOPE | 20%(12/62) |
| DA-CHOPE*^1^ | 5%(3/62) |
| Other single regimen*^2^ | 6%(4/62) |
| Multiple regimens | 21%(13/62) |
| Unknown regimen | 24%(15/62) |
| Outcome |  |
| 0 - 12 months | 46% (29/62) |
| >12 months | 31% (19/62) |
| Alive | 13% (8/62) |
| Unclear/Not report | 10%(6/62) |

*1: DA-CHOPE chemotherapy regimen is an intensified version of the CHOPE regimen with the addition of dose adjustment. *2: Other single regimen: GemOX (Gemcitabine and Oxaliplatin), GOP (Gemcitabine, Oxaliplatin and Paclitaxel, GVD (Gemcitabine, Vinorelbine, Pegylated liposomal doxorubicin) and Anthracyclines.

**Table S2 The blood test results of the patient**

| Laboratory parameter | Case 1 | Case 2 | Case 3 | Normal range |
| --- | --- | --- | --- | --- |
| WBC (×10^9^/L) | 8.88 | 5.84 | 6.81 | 3.5-9.5 |
| PLT (×10^9^/L) | 380 | 417 | 304 | 125-350 |
| HBG (g/L) | 101 | 72 | 66 | 115-150 |
| TP (g/L) | 25.2 | 56 | 38.9 | 65-85 |
| Alb (g/L) | 29.9 | 27.8 | 20.1 | 40-55 |
| ALT (U/L) | 12.1 | 7.8 | 15.8 | 9-50 |
| AST (U/L) | 14.8 | 13.5 | 12.2 | 15-40 |
| CRP (mg/L) | 63.81 | 63.78 | -* | 0-10 |
| SCr (µmol/L) | 73.6 | 38.3 | 60.9 | 57-111 |
| BUN (mmol/L) | 4.4 | 3.3 | 2.9 | 3.6-9.5 |
| LDH (U/L) | 135.5 | 266.8 | 121.7 | 120-250 |
| TBiL (µmol/L) | 15.3 | 8.7 | 6.0 | 0-23 |
| DBiL (µmol/L) | 3.3 | 2.7 | 1.6 | 0-4 |
| IBiL (µmol/L) | 12.0 | 6.0 | 4.4 | - |

* : the patient did not undergo this investigation.


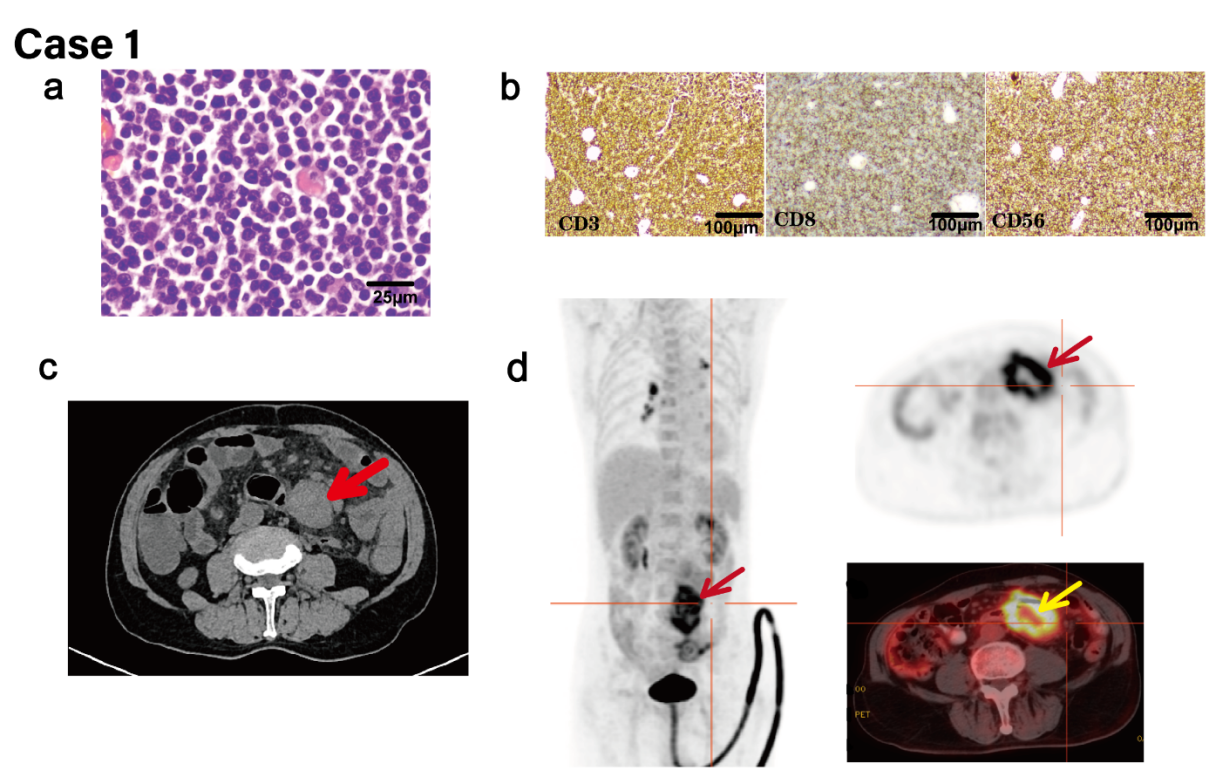


**Fig. S1 The morphologic examination results of Case 1**


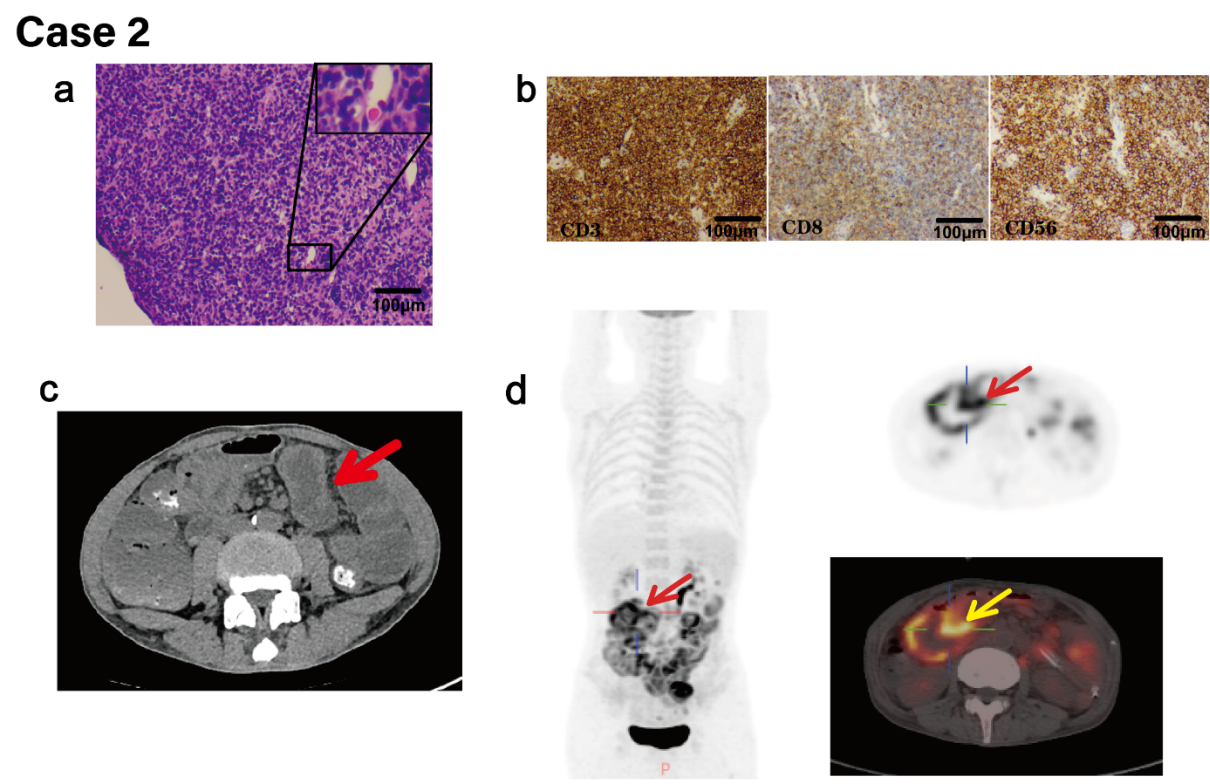


**Fig. S2 The morphologic examination results of Case 2**


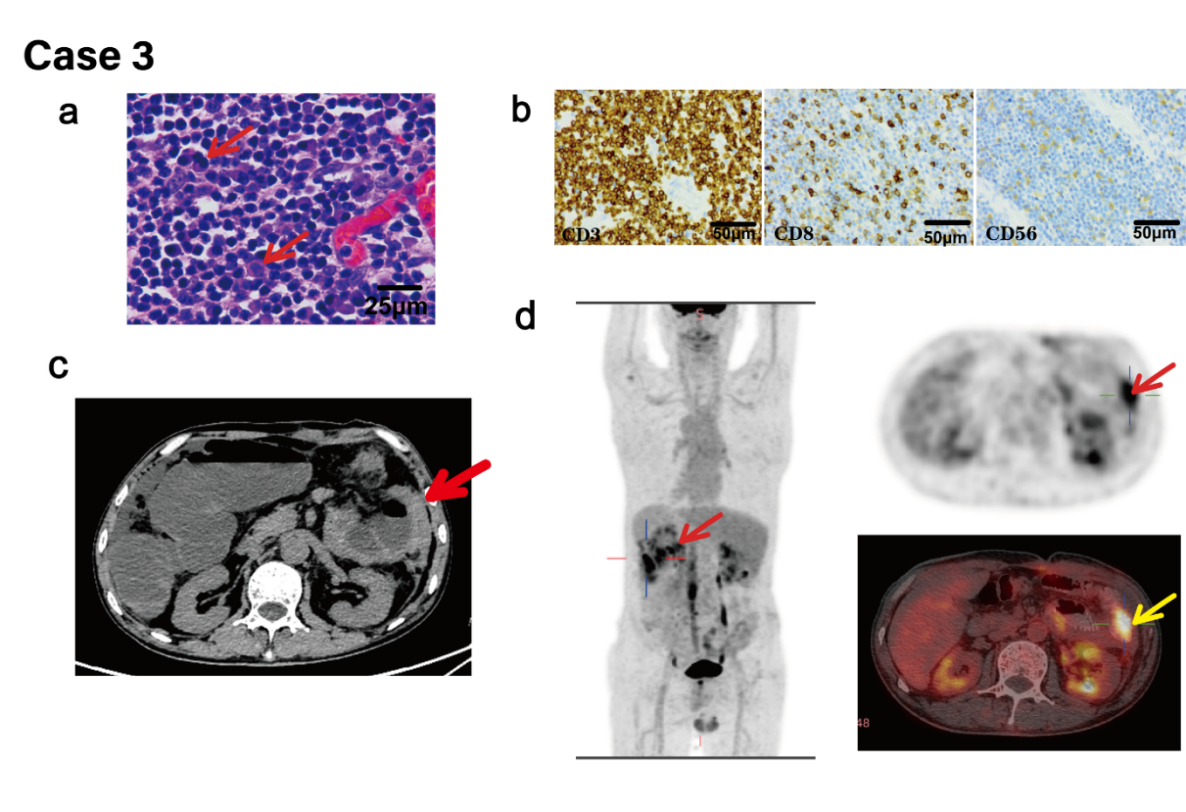


**Fig. S3 The morphologic examination results of Case 3**
